# How is epigenetic information maintained through DNA replication?

**DOI:** 10.1186/1756-8935-6-32

**Published:** 2013-10-02

**Authors:** Varija N Budhavarapu, Myrriah Chavez, Jessica K Tyler

**Affiliations:** 1 Department of Biochemistry and Molecular Biology, University of Texas MD Anderson Cancer Center, 1515 Holcombe Blvd, Houston, TX 77030, USA

**Keywords:** DNA replication, Histone chaperones, Epigenetic factors, Histone modifying enzymes

## Abstract

DNA replication is a highly conserved process that accurately copies the genetic information from one generation to the next. The processes of chromatin disassembly and reassembly during DNA replication also have to be precisely regulated to ensure that the genetic material is compactly packaged to fit into the nucleus while also maintaining the epigenetic information that is carried by the histone proteins bound to the DNA, through cell divisions. Half of the histones that are deposited during replication are from the parental chromatin and carry the parental epigenetic information, while the other half of the histones are newly-synthesized. It has been of growing interest to understand how the parental pattern of epigenetic marks is re-established on the newly-synthesized histones, in a DNA sequence-specific manner, in order to maintain the epigenetic information through cell divisions. In this review we will discuss how histone chaperone proteins precisely coordinate the chromatin assembly process during DNA replication. We also discuss the recent evidence that histone-modifying enzymes, rather than the parental histones, are themselves epigenetic factors that remain associated with the DNA through replication to re-establish the epigenetic information on the newly-assembled chromatin.

## Review

### Introduction

Chromatin is a dynamic structure that controls access by the cellular machineries to the genetic information in a localized manner. Via controlling access to the DNA, chromatin enables the accurate regulation of all genomic processes including DNA repair, DNA replication, and transcription. Chromatin comprises approximately an equivalent mass of DNA and the positively charged histone proteins. Approximately 147 bp of DNA is packaged by an octamer of four core histone proteins (two molecules each of H2A, H2B, H3, H4) to make up the basic repeating unit of chromatin known as the nucleosome [1]. Nucleosomes exist in arrays separated by short histone-free regions called linker DNA. Histone proteins are some of the most evolutionarily conserved proteins in nature and they share a common structural motif known as the histone fold domain, which consists of three alpha helices connected by loops that mediate histone-histone and histone-DNA contacts through the formation of a 4-helix bundle within the H2A-H2B and H3-H4 histone heterodimers [2]. The relatively small but largely hydrophobic contact surfaces within these 4-helix bundles allow reversible assembly of the nucleosome at physiological conditions [3].

The N- and C-terminal tails of the histones protrude out of the globular core of the nucleosome and serve to regulate the function of the chromatin via a wide variety of post-translational modifications on their amino acid side chains which either make the DNA more accessible or less accessible, depending on the precise identity of the post-translational modifications [4]. In effect, the local pattern of post-translational modifications on the histones at any given genomic region carries epigenetic information that serves to regulate the cellular activities that occur on that particular genomic region, for example, its transcriptional activity. However, during DNA replication, the parental histone proteins are all removed from the DNA during the process of chromatin disassembly, and the chromatin is reassembled onto the two daughter DNA duplexes following DNA replication. This raises the question: how are the patterns of post-translational histone modifications that were present on the parental chromatin at each particular DNA sequence re-established or inherited onto the chromatin of the daughter DNA molecules, in order to maintain the localized function of each region of the genome through cell division?

**Figure 1:**
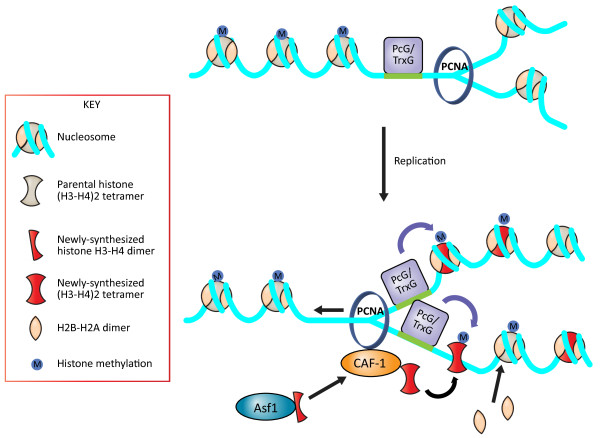
**Model for transfer of epigenetic modifications during DNA replication.** Passage of replication machinery completely removes the parental histones and their marks while retaining certain histone modifying enzymes such as the PcG/TrxG complexes still bound to their DNA elements (top panel). After the passage of the replication fork the histone chaperone ASF1 transfers the newly-synthesized H3-H4 dimer to the histone chaperone CAF-1 which in turn gets recruited to the sites of replication via its binding to PCNA and deposits the H3-H4 tetramer onto the newly-replicated DNA. Once the nucleosome core particle is assembled, adjacent histone modifying enzymes add the specific modification on the histones such as methylation in the above model.

A thorough appreciation of the mechanisms of chromatin disassembly and reassembly during DNA replication may be critical for understanding how the epigenetic information present on the parental chromatin is reinstated on the chromatin of the daughter genomes. Chromatin assembly and disassembly are highly orchestrated processes that are coordinated by histone chaperones and ATP-dependent chromatin remodeling complexes (Figure 1) [5]. Histone chaperones promote chromatin assembly by preventing non-specific histone-DNA interactions while also promoting the correct histone-DNA interactions (reviewed in [6]). Recent studies have begun elucidating the dynamic nature of these histone-chaperone interactions that propose a mechanism of their delivery onto newly-replicated DNA, as discussed below.

### The step-wise process of chromatin assembly

Chromatin assembly is a step-wise process which entails the deposition of the H3-H4 tetramer onto the DNA (or two H3-H4 heterodimers), followed by the deposition of two H2A-H2B dimers flanking the (H3-H4)_2_ tetramer to form the complete nucleosomal core particle [7,8]. However, the histones undergo a complicated and highly coordinated journey *en route* to the DNA. Following their protein synthesis, the newly-synthesized core histone proteins are passed between various different histone chaperones in a highly orchestrated manner [9,10]. The penultimate histone chaperone to receive H3-H4 heterodimers along this journey towards the DNA is Anti-silencing function 1 (Asf1) [11]. Asf1 in turn hands-off the H3-H4 dimers to other histone chaperones that either deposit H3-H4 dimers onto the DNA in a replication-independent manner, such as HIRA [12,13] or histone chaperones that assemble the H3-H4 tetramers onto the DNA in a replication-dependent manner. Whether Asf1 hands-off the histones to a replication-dependent histone chaperone *versus* a replication-independent histone chaperone depends on whether the H3-H4 dimer includes the canonical replication-dependent histone H3 termed H3.1 or the replication-independent histone variant H3.3 [14].

The replication-dependent histone chaperones include Chromatin Assembly Factor 1 (CAF-1) [15] and Rtt106 (at least in yeast) [16]. CAF-1 and Rtt106 each receive two H3-H4 heterodimers from Asf1, from which they facilitate the formation of the H3-H4 tetramer [17-19]. In the next step, the replication-dependent histone chaperones, such as CAF-1, transfer newly-synthesized (H3-H4)_2_ tetramers to the newly-replicated DNA [20] (Figure 1). Currently, our understanding of chromatin assembly after DNA replication, described here, is limited to the incorporation of newly-synthesized histones, which carry their own pattern of deposition-specific histone modifications that are rapidly unmodified following chromatin assembly. These newly-synthesized histones have to somehow gain the parental pattern of histone modifications. Furthermore, the parental histones carrying the parental pattern of post-translational modifications either have to be reassembled back onto the identical DNA sequences on the daughter DNA that they occupied on the parental DNA, or the histone post-translational modifications have to be re-established on the parental histones in a DNA sequence specific manner after DNA replication. The mechanisms by which parental histones are removed from the old DNA and reassembled onto the newly-replicated DNA largely remain a mystery.

### Models for inheritance of histone post-translational modifications through replication

One idea that was briefly favored for the epigenetic inheritance of post-translational histone modifications through replication was that the parental (H3-H4)_2_ tetramer may be split into two H3-H4 dimers [21]. In this scenario, one parental H3-H4 dimer is transferred to each of the newly-replicated DNA molecules, which is joined by a newly-synthesized H3-H4 dimer to complete the (H3-H4)_2_ tetramer, and each parental H3-H4 dimer might then act as a template for reinstating the pattern of post-translational modifications onto the newly-synthesized histones. However, all the evidence indicates that the parental (H3-H4)_2_ tetramer is not split but remains intact during DNA replication [13,22], clearly showing that this idea is wrong. Another possibility for inheritance of histone modifications through replication is that the parental histones carrying the histone-modifications may be reassembled back onto the same DNA sequences on the newly-replicated DNA molecules that they occupied on the parental DNA. These post-translationally modified histones could then potentially template for the modification of adjacent nucleosomes, perhaps by recruiting histone modifying enzymes. While the templating idea is feasible, given that many histone modifiers are recruited by a partner effector protein that recognizes the modified product (reviewed in [23]), it would be technically very challenging to test whether the same histone molecule occupies the identical DNA sequence after DNA replication. If parental histones were reincorporated onto the identical DNA sequences after DNA replication, it would require that cells have a mechanism to physically maintain the parental histones in the immediate vicinity of the DNA replication fork, to promote their reassembly onto the same sequences of the newly-synthesized DNA. Alternatively, the histone modifying enzymes that incorporated the histone-modifications in the first place could be re-recruited to the newly-replicated DNA. Below we discuss examples of histone modifiers being recruited directly or indirectly by the DNA replication machinery, while in other instances, the histone modifiers appear to be recruited by DNA methylation. In both of these later scenarios, clearly some additional levels of regulation would be required in order to re-establish the histone post-translational modification only at the correct regions of the genome rather than broadly.

### Recruitment of histone modifiers to heterochromatin via interaction with the replication machinery

Different parts of the genome carry different histone modifications, which in turn determine the level of compaction and transcriptional activity of different regions of the genome. For example, heterochromatin is characterized by trimethylation of H3K9 in mammals and dimethylation in fission yeast and drosophila, which subsequently recruits the heterochromatin protein 1 (HP1) to coat and condense heterochromatin. The correct histone post-translational modifications, such as H3K9me3, have to be re-established within the heterochromatin domains following DNA replication. The replication-specific histone chaperone CAF-1 plays a key role in the inheritance of H3K9me3 in pericentric heterochromatin regions during DNA replication. CAF-1 is localized to sites of DNA replication through its interaction with the replication protein proliferating cell nuclear antigen (PCNA) [24-26]. CAF-1, in addition to chaperoning histone H3.1-H4, also appears to chaperone HP1 [27], potentially collecting the parental HP1 that is released during DNA replication and acting to sequester it ready for its reincorporation onto the newly-replicated chromatin. CAF-1-HP1 forms a complex with the methyltransferase SETDB1 that monomethylates H3K9 during S phase [28]. The monomethylated H3K9me1 would then presumably act as a substrate for further di- and trimethylation by the SUV39H methyltransferase enzymes, and the resulting H3K9me3 would in turn recruit the HP1 back to the chromatin via the interaction between HP1’s chromodomain and H3K9me3. Furthermore, HP1 binds to SUV39H, acting to recruit SUV39H to the chromatin which presumably methylates adjacent nucleosomes, which would then recruit HP1, leading to the spreading and propagation of the heterochromatin domain [29]. Given that the machinery that is required to re-establish H3K9me3 are localized to replication forks, it is somewhat surprising that the kinetics of H3K9me3 re-establishment is gradual, not rapid, after DNA replication [30]. This suggests that the situation is more complex than it would appear on the surface.

The mechanism for re-establishment of the H3K9me3 in heterochromatin during replication also requires small RNAs that are processed from heterochromatin encoded transcripts. It has been shown in fission yeast that these transcripts are generated preferentially during replication of the heterochromatin-leading strand [31]. Specifically, the Cdc20 subunit of DNA polymerase epsilon promotes the transcription of the pericentric DNA repeats, and the resulting siRNAs promote the localized methylation of H3K9 by Clr4 within the heterochromatin [31]. A similar RNA-guided mechanism for the formation of heterochromatin appears to be occurring in human cells, given that treatment of cells with RNAse destroys both the heterochromatin structure and HP1 localization [32,33].

PCNA also mediates the replication-coupled recruitment of histone deacetylases (HDACs) to the replication fork [34]. The maintenance DNA methylase DNMT1, which is tethered to replication forks via its interaction with PCNA, also recruits the histone methyl transferase G9a during DNA replication [35]. PCNA also recruits chromatin remodelers such as the William Syndrome transcription factor to the sites of replication to in turn associate with the Snf2h subunit of the ISWI complex [36]. As such, there are clear examples of specific histone modifier enzymes, particularly those that generate repressive histone post-translational modifications, being physically recruited to the site of DNA replication to re-establish the histone post-translational modifications [37,38].

### Recruitment of histone modifiers by DNA methylation

The inheritance of DNA methylation through replication occurs readily and rapidly, given that the hypomethylated newly-replicated DNA serves to recruit the maintenance DNA methylases to reinstate DNA methylation on the newly-replicated DNA strand. Furthermore, PCNA helps recruits the maintenance DNA methyltransferase DNMT1 to replication forks [39]. The methylated DNA in turn potentiates the re-establishment of the histone post-translational modification pattern following DNA replication. This is because DNA methylation is recognized by proteins carrying methyl-CpG binding domains (MBDs), which subsequently recruit histone deacetylases and other histone modification proteins. In other words, MBDs form bridges between the methylated DNA and histone modifiers that generate repressive histone post-translational modifications.

MBD1 associates with the H3K9 methyl transferase SUV39H1-HP1 complex to bring about transcriptional repression [40]. MBD1 also associates with the H3K9 monomethyl transferase SETDB1 [28]. Indeed, DNA methylation, via its ability to recruit MBD1, is required for the formation of the SETDB1-CAF-1 complex described above that promotes the H3K9 methylation within pericentric heterochromatin following replication [28].

MBD2 and MBD3 are two interchangeable essential subunits of the NuRD histone deacetylation and ATP-dependent nucleosome remodeling complex [41]. MBD2 and MBD3 bind to the HDAC1 and HDAC2 subunits of NuRD, presumably to promote recruitment of NuRD to methylated DNA. MBD2 and MBD3 are not redundant, but appear to form two functionally distinct NuRD complexes [42], because lack of MBD2 leads to expression of genes that should be normally repressed in the immune system and during X-inactivation [43,44]. Meanwhile, lack of MBD3 leads to persistent expression of undifferentiated cell markers such as *Oct4* and *Nanog* during development causing mouse embryonic lethality [45]. Given that both MBD2 and MBD3 bind to methylated CpG, there must exist further levels of regulation that determine exactly which genes they are recruited to, presumably mediated by additional protein-protein interactions with these complexes. Indeed MBD2 and MBD3 also demonstrate methylation-independent localization on the chromatin [46]. It is important to realize that recruitment of histone modifier enzymes via MBDs binding to methylated DNA would not necessarily be limited to S-phase, as it could occur throughout the cell cycle. However, in the case of NuRD, its recruitment to pericentric heterochromatin is tightly temporally linked to ongoing DNA replication [47]. Furthermore, knockdown of NuRD leads to incomplete assembly of the pericentric heterochromatin and defects in H3K9 trimethylation [48], suggesting that histone deacetylation or chromatin remodeling is a prerequisite for re-establishment of the pericentric heterochromatin after DNA replication.

### Timing of re-establishment of histone modifications after DNA replication

The studies described above provided molecular evidence for histone modifiers being physically recruited to the sites of DNA replication, but they do not answer the questions of how rapidly and how faithfully are the histone post-translational modifications re-established after DNA replication? New methods using quantitative mass spectrometry analysis of stable isotope labeled pre-existing and newly deposited histones has enabled these questions to be answered. This technique has revealed that H4K20me2, a repressive histone modification, progressively accumulates throughout the cell cycle rather than being established following DNA replication [49,50]. In retrospect this result was not too surprising, given that monomethylation of H4K20 is a prerequisite for its dimethylation, and the enzyme that mediates H4K20me1 is only expressed in G2-G1 phases of the cell cycle [51]. Using a similar approach it has been shown that H3K79 methylation patterns are not specifically re-established following DNA replication, but rather occur throughout the cell cycle [52]. In addition use of such stable isotope labeling and mass spectrometry approaches have also shown that the overall histone lysine methylation pattern including H3K9 and H3K27 are transiently reduced during S-phase and are gradually re-established before the onset of the next S-phase [30]. Clearly, these studies indicate that some histone methylation patterns are gradually re-established during the cell cycle in a manner that is independent of DNA replication.

### Dilution of a pre-replicative boost of histone modification to achieve epigenetic inheritance through replication

The Polycomb group (PcG) proteins establish the repressive chromatin mark H3K27me3 in order to control gene silencing transcriptional programs that lock cell identity and memory. Rather than being recruited to the replication fork to re-establish the histone modification, PcG and H3K27me3 accumulate at polycomb response elements (PRE) prior to DNA replication in early S phase [53]. By contrast, these regions are replicated in late S phase by which time PcG levels at the PRE are greatly reduced. These observations suggest that the PcG-dependent H3K27me3 mark is inherited by dilution through replication, rather than by *de novo* methylation occurring at the time of replication. Similarly, H3K4me3, a mark that correlates with transcriptionally active chromatin, was also enriched in early S phase preceding the replication-dependent dilution of this mark [54]. As such, some histone modifications appear to be epigenetically inherited via a pre-replicative boost, which is subsequently diluted during DNA replication. This mechanism has the advantage of: (1) ensuring that very similar sequences within the two newly-replicated DNA molecules obtain the histone modification that was present on the parental DNA, and (2) that the histone modification is absent from that particular DNA sequence for the minimal length of time. As such, the dilution mechanism would ensure accurate and rapid epigenetic inheritance through DNA replication.

### Inheritance of the histone modifier enzymes through DNA replication, even in the absence of histones

A unique situation for H3K27me3 re-establishment appears to occur in *Drosophila* early embryos at the blastomere stage. H3K27me3 is not very abundant at this developmental stage, and rather than diluting the modified histones through replication, it appears that the histones carrying H3K4me3 and H3K27me3 are replaced by unmethylated H3 following DNA replication [55]. Indeed, these methylation marks could not even be detected in S phase nuclei of blastomere stage *Drosophila* early embryos. This is in contrast to the situation in mammalian cells where H3K27me3 has a long half-life and is readily detected during S phase [56]. In *Drosophila* early embryos at the blastomere stage, the PcG proteins that mediate H3K27me3 and the Trithorax group (TrxG) proteins that mediate H3K4me3 continuously associate with their DNA binding elements throughout replication. This result suggests that PcG and TrxG re-establish the histone modifications onto newly-assembled unmethylated histones. This work demonstrates that PcG and TrxG proteins, rather than the modified histones themselves, are the epigenetic marks that are inherited through DNA replication, at least during this specific developmental stage of *Drosophila* development (Figure 1). Biochemical experiments provide support for the idea that DNA-bound PcG proteins are inherited through DNA replication [57]. This work used recombinant chromatin templates replicated in an *in vitro* SV40 replication system by HeLa cell extracts supplemented with *Xenopus* egg extract fractions enriched with the histone chaperone nucleoplasmin. In this system, polycomb repressive complex 1 (PRC1)-group proteins remained bound to chromatin and DNA throughout the replication process. PRC1 persisted on the DNA during replication fork passage and H3K27me3 was not required to maintain PRC1 on DNA during replication.

The biggest challenge for this hypothesis is understanding how these histone modifying enzymes are retained on the DNA during replication. The presence of preSET domains in Trx and the Ez subunit of PRC1 might facilitate their binding to ssDNA during DNA unwinding ahead of the replication fork [58]. However, the precise mechanism of how these proteins are transferred back to the nascent DNA needs to be still elucidated. In a set of recent papers, the Francis group has shown that each PRC1 complex can stoichiometrically bind to one nucleosome and one other PRC1 complex such that PRC1 can be retained on chromatin due to its ability to bind to both nucleosomes and self, leading to bridging of nucleosomes resulting in oligomeric structures [59,60]. They have demonstrated that PRC1-PRC1 interactions help in holding the PRC1 complex in position while the transient dissociation of PRC1-chromatin interactions facilitates the passage of the replication fork. These studies indicate that the histone modifying enzymes can be the actual epigenetic marks in contrast to the modified histones themselves being the epigenetic marks.

## Conclusions

By contrast to the single mechanism for copying genetic information by semi-conservative replication, recent studies suggest that copying of the epigenetic information is a lot more complicated and varied. In some cases, such as the dilution model, the histone modifications do indeed appear to be directly inherited from the parental chromatin. In other instances, distinct mechanisms exist to re-establish different histone marks after DNA replication. In some cases, the histone-modifying enzyme is recruited to the replication fork, while in other cases the histone-modifying enzyme itself is maintained on the DNA through DNA replication. In other cases, the histone modifications are re-established in a much less immediate manner throughout the cell cycle. Although not mutually exclusive, sequence-specific DNA binding factors also presumably re-recruit histone modifiers to the chromatin to re-establish histone modification patterns. Presumably the mechanism that is used to inherit or re-establish each histone post-translational modification depends on the immediacy and accuracy required by the cell for the presence of that particular epigenetic mark.

## Abbreviations

ASF1: Anti-silencing Function 1; CAF1: Chromatin assembly factor 1; Cdc20: Cell division cycle 20; DNMT1: DNA (cytosine-5)-methyltransferase 1; H3 K9me3: Trimethylated Histone 3 at Lysine 9; H4 K20me2: Dimethylated Histone 4 at Lysine 20; H3 K27me3: Trimethylated Histone 3 at Lysine 27; H3 K4me3: Trimethylated Hisotne 3 at Lysine 4; NuRD: Nucleosome remodeling and histone deacetylase; PRC1: Polycomb-group repressive complex 1; SETDB1: SET domain, bifurcated 1; SUV39H: Suppressor of variegation 3–9 homolog 1.

## Competing interests

The authors declare that they have no competing interests.

## Authors’ contributions

VNB and MSC contributed equally to the writing of the manuscript and making the figure. JKT edited the manuscript. All authors read and approved the final manuscript.
